# Esketamine nasal spray: real world use in the Department of Mental Health of Rimini (Italy)

**DOI:** 10.1192/j.eurpsy.2026.11770

**Published:** 2026-07-31

**Authors:** M. P. Rapagnani, L. Veronesi, F. Sartini

**Affiliations:** Psychiatry, Ausl della Romagna, Rimini, Italy

## Abstract

**Introduction:**

In treatment-resistant depression, commonly defined as a lack of response to two or more consecutive treatments of antidepressants. Esketamine (the S-enantiomer of ketamine) nasal spray, administered in combination with either a selective serotonin reuptake inhibitor (SSRI) or a serotonine-norepinephrine reuptake inhibitor (SNRI), is the only treatment approved in Europe specifically for treatment-resistant depression.

**Objectives:**

The aim of the study is to evaluate the effectiveness and safety of esketamine nasal spray in a clinical sample of patients with TRD in the Department of Mental Health of Rimini, in a real world setting, no exclusion criteria.

**Methods:**

We retrospectively reviewed medical records of patients, from November 2023 (first patient to have the esketamine nasal spray therapy in the Department) to August 2025, inpatients ed outpatients, that have more than 18 years, who received the esketamine nasal spray (Spravato®). We valuated retrospectively the days of hospitalization after the administration, the number of emergency room accesses (ER), the tolerability and the efficacy analyzing the “repository of AUSL della Romagna”, the discharge letter from the Psychiatric Hospital and the “CURE” program of the Psychiatric Service of Rimini.

**Results:**

We enrolled 10 patients, 7 males and 3 females. Average age 48.6. Two males patients started the treatment in the Psychiatric Hospital, others as outpatients. All patients had a long story of depression with use, over the years, of multiple antidepressants. In addition at the TRD diagnosis, the others comorbidity diagnosis were: 2 pt mood disorder (included bipolar dorder), 1 pt ciclotimia, 1 pt avoidant personality disorder, 1 pt Asperger’s syndrome, 1 pt autism, 1 pt borderline personality disorder, 1 pt psychotic depression and 1 pt recurrent depressive disorder. Four patients don’t have finished the six months treatment.. The pt with diagnosis of BPD drop-out after three months, but didn’t has no longer admission in the psychiatric hospital. Dosages are 56 mg or 84 mg. Antidepressant in combination are SSRI, SNRI and multimodal (vortioxetine). One pt had two episodes of increased pressure with no access in the emergency-room, but needed one time of antihypertensive treatment, another time no treatment; one pt one episode of dissociation. No others side effects, no hospitalization or ER accesses. The pt who finished the tratment are in good psychiatric health.

**Conclusions:**

Our observational data support the safety and tolerability of esketamine in a real-world TRD sample. Our data also indicate clinical effecacy of esketamine nasal spray in this population, even if a small sample. There were no evidence of abuse, misuse, withdrawal, and no longer term cognitive or urogenital or hepatic toxicity.

**Disclosure of Interest:**

None Declared

